# The role of clinically-relevant parameters on the cohesiveness of sclerosing foams in a biomimetic vein model

**DOI:** 10.1007/s10856-015-5587-z

**Published:** 2015-10-08

**Authors:** Dario Carugo, Dyan N. Ankrett, Vincent O’Byrne, David D. I. Wright, Andrew L. Lewis, Martyn Hill, Xunli Zhang

**Affiliations:** Bioengineering Science Research Group, Faculty of Engineering and the Environment, University of Southampton, Southampton, SO17 1BJ UK; Electro-Mechanical Engineering Research Group, Faculty of Engineering and the Environment, University of Southampton, Southampton, SO17 1BJ UK; Biocompatibles UK Ltd. A BTG International Group Company, Farnham Business Park, Weydon Lane, Farnham, Surrey, GU9 8QL UK; BTG International Ltd., 5 Fleet Place, London, EC4M 7RD UK; Institute for Life Sciences, University of Southampton, Southampton, SO17 1BJ UK

## Abstract

We have recently reported on the development of a biomimetic vein model to measure the performance of sclerosing foams. In this study we employed the model to compare the commercially-available Varithena^®^ (polidocanol injectable foam) 1 % varicose vein treatment (referred to as polidocanol endovenous microfoam, or PEM) with physician compounded foams (PCFs) made using different foam generation methods (Double Syringe System and Tessari methods) and different foam formulations [liquid to gas ratios of 1:3 or 1:7; gas mixtures composed of 100 % CO_2_, various CO_2_:O_2_ mixtures and room air (RA)]. PCFs produced using the DSS method had longer dwell times (DTs) (range 0.54–2.21 s/cm in the 4 mm diameter vein model) than those of the corresponding PCFs produced by the Tessari technique (range 0.29–0.94 s/cm). PEM had the longest DT indicating the best cohesive stability of any of the foams produced (2.92 s/cm). Other biomimetic model variables investigated included effect of vessel size, delayed injection and rate of plug formation (injection speed). When comparing the 4 and 10 mm vessel diameters, the DTs seen in the 10 mm vessel were higher than those observed for the 4 mm vessel, as the vein angle had been reduced to 5° to allow for foam plug formation. PCF foam performance was in the order RA > CO_2_:O_2_ (35:65) ≅ CO_2_:O_2_ (65:35) > CO_2_; PEM had a longer DT than all PCFs (22.10 s/cm) except that for RA made by DSS which was similar but more variable. The effect of delayed injection was also investigated and the DT for PEM remained the longest of all foams with the lowest percentage deviation with respect to the mean values, indicating a consistent foam performance. When considering rate of plug formation, PEM consistently produced the longest DTs and this was possible even at low plug expansion rates (mean 29.5 mm/s, minimum 20.9 mm/s). The developed vein model has therefore demonstrated that PEM consistently displays higher foam stability and cohesiveness when compared to PCFs, over a range of clinically-relevant operational variables.

**Electronic supplementary material:**

The online version of this article (doi:10.1007/s10856-015-5587-z) contains supplementary material, which is available to authorized users.

## Introduction

Varicose veins are a very common disease with symptomatology that can significantly impact on a patient’s Quality of Life (QoL) and—if left untreated—can cause long-term injury to the skin, resulting in discolouration, inflammation, and ultimately ulceration [1]. Vein varicosities are related to congential or acquired abnormalities of the deep venous system and venous valves, or vein wall weakness [2]. Although life-threatening complications are rare, there is a considerable social and economic impact of vein disease [3].

Several methods have been developed to treat varicose veins, including radiofrequency and laser ablation, venous stripping, surgery and sclerotherapy [4]. Sclerotherapy is the least invasive method, and involves the intra-venous injection of a liquid sclerosing agent (i.e., sodium tetradecyl sulphate or polidocanol) to disrupt the endothelial lining of the vein and ultimately lead to vessel sclerosis [5]. However, the use of liquid sclerotherapy is confined to smaller veins (i.e., ≤3 mm in diameter) [6], as the liquid sclerosant is diluted and deactivated rapidly by blood [7]. In order to overcome this limitation, foamed sclerosants have been introduced, which displace rather than mix with blood, leading to greater contact time with the vein endothelium [8]. Foamed sclerosants are usually produced manually by the clinician, referred to as physician compounded foams (PCFs). The two major techniques of PCF production are (i) the double syringe system (DSS) method [9], and (ii) the Tessari method [10]. In these techniques a gaseous and a liquid phase are manually mixed by connecting two syringes together using a Combydin adapter (DSS) or a three-way tap (Tessari). PCF created using room air (RA) has been widely used, although the nitrogen (N_2_) content that contributes to its stability [11, 12] also increases the risk of gas embolism, with chest tightness being reported as well as neurological symptoms [13]. This has prompted investigation into foams made using clinical grade carbon dioxide (CO_2_), or CO_2_ and oxygen (O_2_) mixtures [14–16]. CO_2_ has greater solubility in blood [17], which means the risk of gas embolism is reduced. These foams are less stable [11], however, and are therefore less efficient at displacing blood [12]. With the aim of improving foam stability and production consistency, automated systems have been recently proposed as an alternative to manual techniques [6, 12, 18, 19]. Among these, Polidocanol Endovenous Microfoam [PEM from herein, manufactured by Provensis Ltd and cleared in the USA under the name Varithena^®^ (polidocanol injectable foam) 1 %] is a proprietary, low nitrogen, CO_2_:O_2_ (35:65) pharmaceutical-grade polidocanol microfoam delivered from a proprietary canister system.

Despite sclerosing foams having been used in the clinical environment [20], a limited amount of work has been conducted to physically characterise foam properties in vitro, particularly under clinically relevant experimental conditions. A typical characterisation of foam properties involves the measurement of bubble size, bubble size distribution, and foam half time (FHT, defined as the time taken for the foam volume to decrease by a factor of two) [21, 22]. However, these parameters are not sufficient to fully characterise the behaviour of sclerosing foams in a clinically relevant scenario. This will depend on the interplay between (i) gravitational effects (i.e., further depending on patient’s leg elevation), (ii) physical properties of the gaseous and liquid phases constituting the foam, (iii) physical properties of the surrounding fluid medium, (iv) bubbles size and size distribution, and (v) clinical factors, such as time delay between foam production and injection, and foam injection rate.

In our previous study we reported on the development of a novel biomimetic model to experimentally measure the interrelated properties of foam-induced liquid displacement and foam cohesiveness [12]. By coupling the model with a computational-based image analysis software, we revealed the dynamics of foam plug degradation in a phantom vein [12]. Importantly, we introduced a novel parameter [Degradation rate (DR)] to quantify and compare the cohesiveness of sclerosing foams [12]. In this study, we employed the developed model to compare the cohesiveness of polidocanol-based PCFs and PEM, and evaluate the effect of clinically-relevant parameters on foam cohesiveness. These include (i) the diameter of the target vessel, (ii) foam production technique (PCF vs. PEM), (iii) type of gaseous phase, (iv) foam density [i.e., liquid:gas ratio (LGR)], (v) time delay between foam production and injection and (vi) foam plug formation rate (injection speed).

Results from this study may provide valuable information to clinicians regarding the choice of the optimal clinical practice to achieve the desired foam cohesiveness and therapeutic effectiveness.

## Materials and methods

### Vein model set-up

**Fig. 1:**
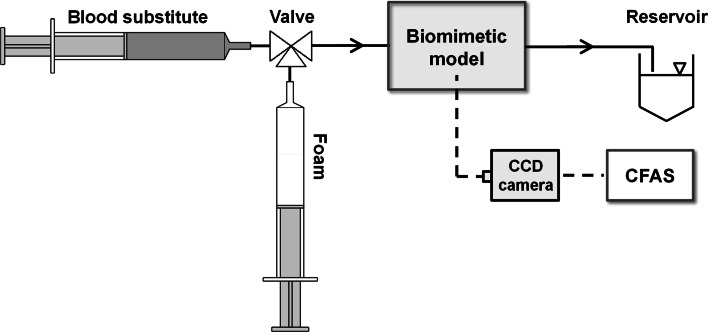
Schematic depiction of the experimental set-up (readapted from Ref. [32]). The biomimetic vein model was initially primed with a blood substitute. Subsequently, sclerosing foam was injected into the model and videos of foam plug expansion and degradation were recorded by a CCD camera. Videos were transferred to the computational foam analysis system (CFAS) for determination of foam plug degradation rate (DR) and dwell time (DT). A three-way valve was used to manually switch between blood substitute and foam. Fluids were discharged in a reservoir

The biomimetic vein model has been described in our previous study [12]. It consists of a segment of polytetrafluoroethylene (PTFE) tubing (Thermo Scientific Inc., USA) positioned in a bespoke platform, which inclination angle (*α*) could be adjusted and measured by a digital liquid–crystal display (LCD) inclinometer (RS Components Ltd., UK) (Fig. 1). 4 and 10 mm inner diameter (ID) tubing were employed in the present investigation, in order to simulate both small and large varicosities [2]. *α* was set to 25° and 5° for the 4 and 10 mm ID tubing, respectively. Fluids and foams were injected into the model via a three-way stopcock (Baxter, USA).

Videos of foam plug expansion and degradation were captured by means of a 1412 × 1059 pixels × pixels high speed charge-coupled device (CCD) camera (Lumix, Panasonic Corporation, Japan), with interframe time interval of 30 ms. The camera was positioned on a metal stand, which height could be adjusted in order to control the positioning of the field of view.

### Experimental protocols

#### Foam production

**Fig. 2:**
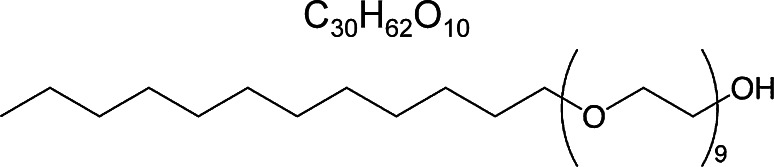
Generic chemical structure of polidocanol. It comprises a mixture of polyethylene glycol monododecyl ethers, averaging nine ethylene oxide groups per molecule

*Physician compounded foams (PCFs)* Polidocanol was employed as a foaming agent throughout these studies (see Fig. 2 for its chemical structure) and was supplied by Croda (Goole, UK) as a white waxy solid with a purity of ≥99.0 %, as determined by gas chromatography. It has a low reported critical micelle concentration of 0.002 % in water or saline [23].

Foams were produced by mixing controlled volumes of the aqueous buffered polidocanol solution and a gas or gas mixture. A polidocanol concentration of 1 % (v/v in buffered saline) was chosen, as clinical studies in varicose vein treatment comparing efficacy of 1 and 3 % polidocanol found no statistically significant difference between the two concentrations [24, 25], and this allowed for direct comparison of PCFs with PEM which is made from 1 % polidocanol. Gas formulations studied included RA, CO_2_ and mixtures of carbon dioxide and oxygen (CO_2_:O_2_). The volume ratio between liquid and gas was varied in a range of clinical interest [26–29], from 1:4 (wet foams) to 1:7 (dry foams).

Two methods of PCF production were investigated in the present study: (i) DSS and (ii) Tessari [30].

**Fig. 3:**
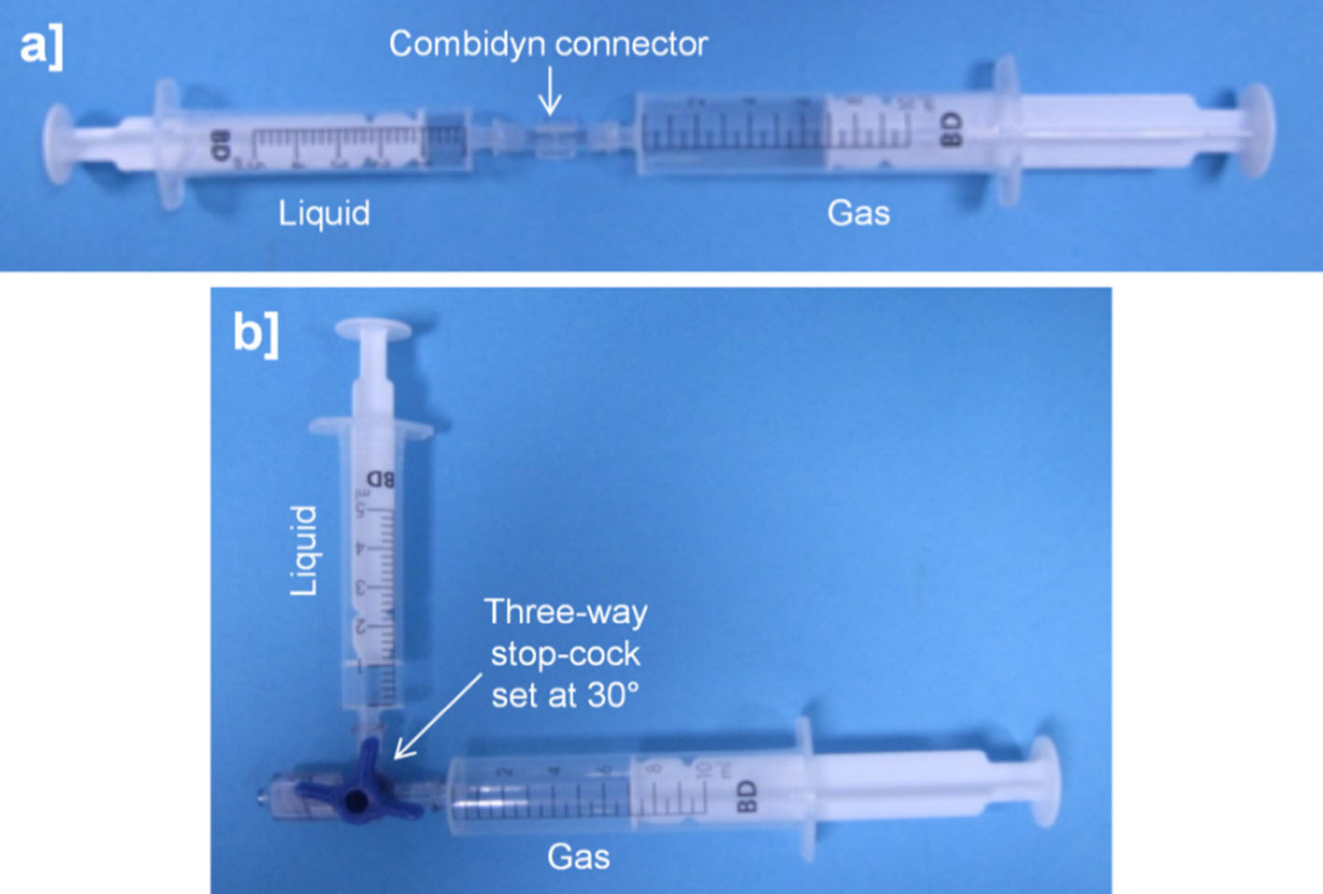
Manual techniques for producing sclerosing foams. In the double syringe system (DSS) method syringes (BD Discardit™ II) were connected by a Combidyn^®^ adapter (**a**), while in the Tessari method they were connected by a three-way valve set at a 30° off-set (**b**). In both production methods, the foam was generated by passing the polidocanol solution (liquid phase) from one syringe, ten times into and out of the other syringe initially containing a gas or gas mixture (gaseous phase). Throughout these studies, foam was produced at room temperature by a single operator

In the DSS method, foam was produced by passing the polidocanol solution from a 5 mL syringe, ten times into and out of a 10 mL syringe. Syringes were purchased from BD Biosciences (USA) and were connected via a Combidyn™ adapter (B. Braun Melsungen, Germany) (Fig. 3a) [30]. In the Tessari method, the straight connector was replaced with a three-way valve which was set at a 30° off-set (Fig. 3b) [30]. Experiments were conducted at room temperature (~23 °C), after foam production, and foams were produced by the same operator.

*Polidocanol endovenous microfoam (PEM) * Varithena^®^ (polidocanol injectable foam) 1 % (denoted as PEM for brevity) is a combination drug device product manufactured by Provensis Ltd (a BTG International group company, London, UK) consisting of a proprietary 35:65 CO_2_:O_2_ gas mixture with ultralow nitrogen content (<0.8 %) and 1 % polidocanol solution, contained within a pressurised canister and combined on discharge from the canister as a uniform microfoam. PEM has a fixed LGR of 1:7. Sterile canisters of the product were employed as per the instructions for use (see Supplementaty Fig. S1). Experiments were conducted at room temperature (~23 °C), after foam production, and foams were produced by the same operator.

#### Measuring foam injection and degradation dynamics

The biomimetic model was filled with a solution of glycerol (Sigma Aldrich Co., USA) in purified water (Milli-Q, Millipore Co., USA), acting as a blood substitute. The volume concentration of glycerol in water was equal to ~30 %, leading to a dynamic viscosity (*μ*) of ~0.003 Pa s and a density (*ρ*) of ~1078 kg/m^3^ [31], simulating the bulk physical properties of blood.

After production, the foam was injected into the model where it formed a plug (see Supplementary Video 1 for a representative experimental injection procedure). The bolus of foam displaced the blood substitute as it travelled upwards (*foam plug expansion phase*). However, the foam plug was transient in nature and receded towards the initial injection site (*foam plug degradation phase*), until complete plug degradation (see Supplementary Video 1).

Foam dynamics were captured by the CCD camera, and videos were transferred to a PC for analysis.

#### Computational foam analysis system

The computational foam analysis system (CFAS v1.0) has been employed to quantify foam cohesiveness, as described in our previous study [12]. Briefly, the software reads the acquired videos and measures the temporal evolution of foam plug length (*L*, in mm), during both the expansion and degradation phases. Finally, it calculates the rate of foam plug degradation [degradation rate (DR) in mm/s] from linear interpolation of the experimental data points during the plug degradation phase. Lower DR as a result of greater foam cohesiveness was taken as an indicator of better clinical performance. Dwell time (DT, in s/cm) is a more clinically meaningful expression of degradation rate as it represents the amount of time that the foam is in contact with the vein wall and can act on the endothelium. DT is derived from DR and is calculated as the inverse of the DR. Supplementary Videos 1–3 show examples of experimental foam plug expansion and degradation in the biomimetic model for different foam formulations, and the corresponding dynamic plot of foam plug length determined by CFAS. DR and DT were determined from this plot, in an automated fashion.

#### Multi-parametric analysis of foam cohesiveness

The effect of clinically-relevant parameters on foam cohesiveness was investigated using the biomimetic model. These are summarised in Table 1, and include:

**Table 1:** Clinically-relevant parameters investigated in the present study, using the developed biomimetic model and computational foam analysis system (CFAS). N = 4 for each experimental condition

Tube ID	4 mm		10 mm	
Foam production technique	DSS	Tessari		PEM
Gaseous phase	RA	35:65 CO_2_:O_2_	65:35 CO_2_:O_2_	100 CO_2_
Liquid:Gas ratio (LGR)	1:3	1:4	1:5	1:7
Time delay before injection	3–5 s		75 s	
Foam plug expansion rate (injection speed)	In the range 20.9–52.1 mm/s

- *Model diameter* 4 and 10 mm ID tubing were employed to simulate both small and large varicose veins.
- *Foam production technique* Foam was produced manually (by DSS or Tessari method), or using PEM.
- *Gaseous phase* Gases investigated for PCF production included RA, 100 % CO_2_, 65 %:35 % CO_2_:O_2_ and 35 %:65 % CO_2_:O_2_.
- *Foam density* Foam density was varied by adjusting the percentage volume of liquid and gaseous phases. LGRs investigated included 1:3, 1:4, 1:5, and 1:7.
- *Time delay between foam preparation and injection in the model* Foam was injected within 3–5 s from production (e.g. immediate injection) or 75 s from production (e.g. delayed injection), to simulate different clinical practises.
- *Effect of injection speed* Foam was injected into the model at different speeds, and the effect on DT determined.

### Measuring foam bubble size distribution

A QICPIC particle size and shape analyser (supplied by Sympatec UK, Bury, Lancashire) was employed to measure bubble size distribution of different foam formulations (see also Ref. [32] for additional information about this measurement system).

For this purpose, a foam sample was loaded within a 10 mL capacity BD syringe and placed on a syringe pump (Harvard Apparatus PHD/ULTRA, Holliston, MA). The foam was injected at a rate of 37.6 mL/min into a stream of deionised water (driven by a peristaltic pump, Watson Marlow 505S, Falmouth, UK) that carried the bubbles through a 2 mm cuvette within the particle analyser where detection occurred. The detector was positioned at the midpoint across the height of the cuvette. A built-in image analysis software captured images of the flowing bubbles at 25 frames per second, and generated a plot of bubble size distribution. Each analysis comprised of five replicates, each consisting of 15 s long acquisition intervals. The time taken from filling of the syringe with foam to beginning of the analysis was ~35–40 s.

## Results

### Effect of method and composition of foam formulation

**Fig. 4:**
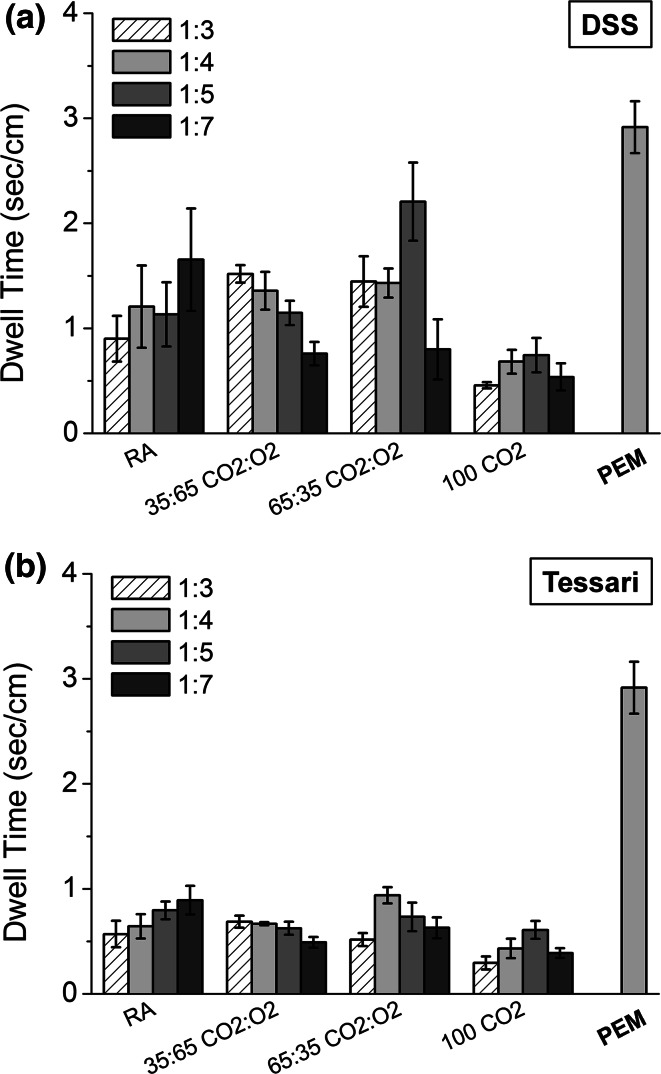
Dwell Times (in s/cm) for DSS PCFs (**a**) or Tessari PCFs (**b**) of different gas formulations and LGRs compared to PEM, in the 4 mm diameter vein model. *DSS* Double syringe system, *PCF* physician compounded foam, *PEM* polidocanol endovenous microfoam, *LGR* liquid to gas ratio

PCFs produced using the DSS method had longer DT in the 4 mm diameter vein model (range 0.54–2.21 s/cm) than those of the corresponding PCFs produced by the Tessari technique (range 0.29–0.94 s/cm) (Fig. 4a vs. b). There was no obvious dependence of the LGR on the DT of any of the PCFs produced using either technique. Foams made using 100 % CO_2_ were less stable, with lower DT than the other PCFs (see Supplementary Video S1). PEM had the longest DT indicating the best cohesive stability of any of the foams produced (DT = 2.92 s/cm), including those PCFs generated using the equivalent LGR (1:7) (see Supplementary Video S2 for a RA PCF, and Video S3 for PEM).

### Effect of biomimetic vein model variables

#### Influence of vessel diameter

**Fig. 5:**
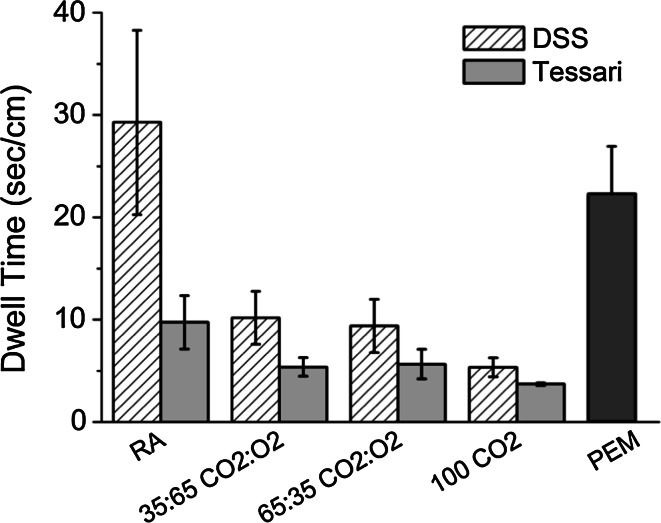
Dwell Times (DT, in s/cm) generated using the 10 mm vein model at 5° inclination angle for DSS PCFs or Tessari PCFs of different gas formulations, compared to PEM. Foams were produced at a fixed LGR = 1:7. *DSS* Double syringe system, *PCF* physician compounded foam, *PEM* polidocanol endovenous microfoam, *LGR* liquid to gas ratio

All foams failed to form a stable foam plug that could displace the blood substitute in the 10 mm diameter vessel, when the angle of inclination was 25°. The angle had to be adjusted by trial and error down to 5° in order to permit the least stable foam (100 % CO_2_ PCF) to create a plug in the vessel for which the DT could be measured. All foam formulations evaluated in the 10 mm diameter vein model had DTs significantly higher than the equivalent formulations used in the 4 mm diameter vein model (Fig. 5), as the vein angle had been reduced. Again, PCFs generated using the DSS method had longer DTs (range 5.3–29.3 s/cm) than the equivalent formulations made using the Tessari technique (range 3.71–9.73 s/cm). PCF foam performance was in the order RA > CO_2_:O_2_ (35:65) ≅ CO_2_:O_2_ (65:35) > CO_2_; PEM had a longer DT than all PCFs (DT = 22.10 s/cm) except that for RA made by DSS which was similar but more variable.

#### Influence of injection delay

**Fig. 6:**
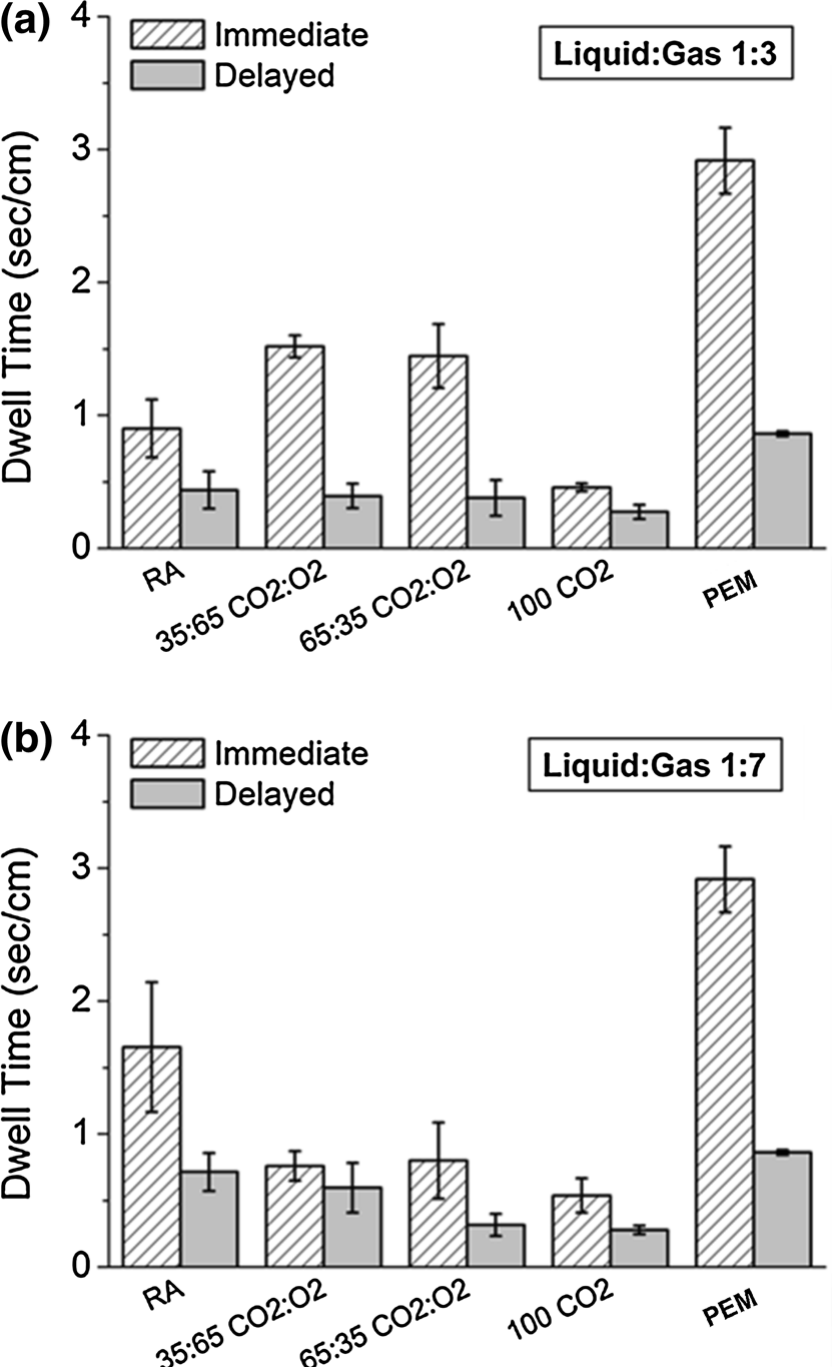
Effect of immediate (3–5 s) versus delayed (75 s) injection on the Dwell Time (DT, in s/cm) for DSS PCFs of different gas formulations made at 1:3 (**a**) or 1:7 (**b**) LGRs compared to PEM. *DSS* Double syringe system, *PCF* physician compounded foam, *PEM* polidocanol endovenous microfoam, *LGR* liquid to gas ratio

PCFs with different gas formulations were manufactured at a typical clinical LGR of 1:3 and also at 1:7 to allow for direct comparison with PEM. Foams were evaluated in the vein model with an intention to determine the influence of injection delay on foam cohesiveness (Fig. 6). CO_2_:O_2_ PCF formulations made with a LGR of 1:3 had longer DT compared to PCFs with a LGR of 1:7, whereas the reverse was true for RA PCFs. These differences between formulations were somewhat lost when injection was delayed (i.e., 75 s from production) with all foams having lower DT compared to those injected immediately (i.e., 3–5 s from production). PEM had the longest DT of all foams with the lowest percentage deviation in DT with respect to the mean values, indicating a consistent foam performance (Table 2), although the PCF with CO_2_:O_2_ (35:65) was also reasonably consistent when injected immediately, which has the same gas mixture formulation as PEM.

**Table 2:** Variability in dwell time (DT) from different foam formulations, expressed as percentage deviation of the mean

	% Deviation of the mean DT 1:3 LGR		% Deviation of the mean DT 1:7 LGR	
	Immediate injection	Delayed injection	Immediate injection	Delayed injection
RA	24.3	32.2	29.6	20.0
35:65 CO_2_:O_2_	5.5	23.1	14.6	31.3
65:35 CO_2_:O_2_	16.7	35.6	35.9	26.5
100 CO_2_	11.8	19.5	24.0	11.8
PEM	8.5	2.2	8.5	2.2

#### Influence of rate of foam plug formation (injection speed)

**Fig. 7:**
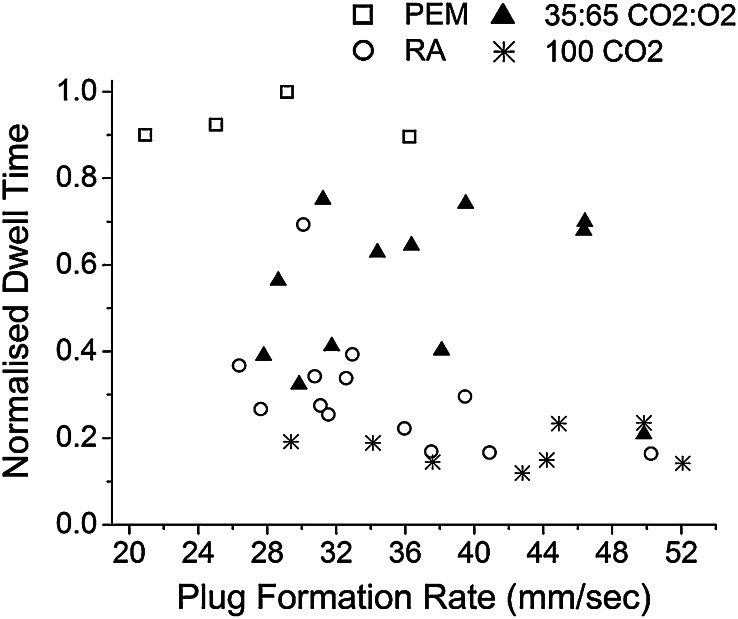
Plot of normalised dwell time (DT) versus Plug Formation Rate (in mm/s) for various PCF formulations (DSS) compared to PEM (foams were injected immediately after production). *DSS* Double syringe system, *PCF* physician compounded foam, *PEM* polidocanol endovenous microfoam, *LGR* liquid to gas ratio

It was observed that it was necessary to inject the various foam formulations into the vein model at different rates in order to ensure a foam plug was formed that would displace the blood substitute. This pseudo-injection speed actually represents the rate of foam formation in the vessel where it volumetrically expands to form a coherent plug. The foam plug formation rate was determined computationally using the CFAS, and was calculated from the gradient of the line interpolating the experimental foam plug length (*L*) vs. time (t) data points (see Supplementary Videos 1–3). Figure 7 shows a plot of the DT (normalised to the highest DT in order to visualise all of the results easily on the same graph) against the plug formation rate required for each experiment, for a selection of PCFs created using the DSS method and PEM. Firstly, it is clear that for the unstable 100 % CO_2_-based foam formulations, the DT is low regardless of the rate of plug expansion, although rates of >50 mm/s were required in some instances to form a suitable foam plug (mean formation rate = 43.7 mm/s). There appears to be no obvious correlation between DT and rate of plug formation for any of the foams (mean formation rate = 35.5 mm/s for RA and 37.7 mm/s for 35:65 CO_2_:O_2_) but what is clear is that PEM consistently produces the longest DT, and this is possible even at low plug formation rates (mean formation rate = 29.5 mm/s). The stability and cohesive properties of the PEM mean that a foam plug can be formed even if the foam is injected relatively slowly (as low as 20.9 mm/s in this series of experiments), whereas for PCFs slow injection will tend to lead to bubble streaming from the leading edge of the foam hindering complete plug formation.

#### Bubble size and size distribution

**Fig. 8:**
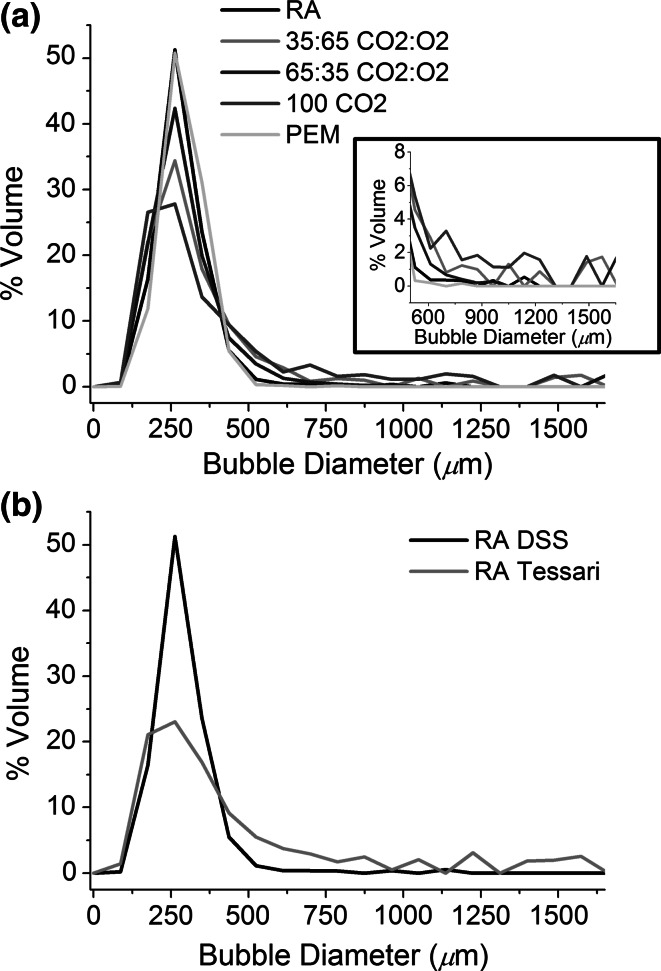
**a** Bubble size distributions (expressed in terms of volume fraction) of DSS PCFs and PEM, obtained using the Sympatec particle size analyser, at a fixed LGR = 1:7 (n = 5). The *inset* shows an expanded view of bubble size distribution for bubble diameters ranging from 550 to 1550 µm. **b** Bubble size distributions of RA DSS versus RA Tessari, at a fixed LGR = 1:7 (n = 5). All measurements in (**a**) and (**b**) were performed 35–40 s after foam production. *DSS* Double syringe system, *PCF* physician compounded foam, *PEM* polidocanol endovenous microfoam, *LGR* liquid to gas ratio

Figure 8 shows bubble size distribution (expressed in terms of volume fraction) of (a) DSS PCFs versus PEM (at a fixed LGR = 1:7) and (b) DSS RA versus Tessari RA. These were measured using the Sympatec particle size analyser, at 35–40 s after foam production.

PCF produced using 100 % CO_2_ had the broadest bubble size distribution, with large bubbles (≫500 μm in diameter) present (see inset in Fig. 8a for a clear view). PCFs produced using mixtures of CO_2_ and O_2_ had a narrower bubble size distribution, but bubbles larger than 500 μm in diameter still persisted in the bubble population. Notably, PEM had a narrower bubble size distribution compared to PCFs with analogous composition, with no large bubbles (i.e., bubble diameter was <~500 μm) which was comparable to RA PCFs (Fig. 8a). The Tessari method produced bubbles which were larger and more polydisperse in size compared to DSS with equivalent composition, as clearly visible from Fig. 8b for RA foams.

## Discussion

When considering the general experimental observations made during this series of studies, it can be seen that PCFs generated using the DSS technique were more stable and produced longer DT in the vein model compared to the same PCFs made by the Tessari method. We have observed this effect before, and have attributed this to the broader bubble size distribution in Tessari PCFs compared to DSS PCFs [32] (Fig. 8). Notably, it has been previously reported that (i) a more uniform bubble size distribution is associated with more stable foams [33, 34], as differences in Laplace pressure among bubbles of different size drive transfer of gas from the smaller to the larger bubbles through the liquid separating them (a process known as disproportionation or Ostwald ripening [35, 36]). This process is further enhanced when the amount of separating liquid reduces [21]. (ii) Foams with smaller bubbles have greater stability, which has been attributed to reduced liquid drainage [37].

CO_2_-containing PCFs are also less stable compared to RA PCFs as they contain a high fraction of a very aqueous-soluble gas (CO_2_) compared to the low solubility of nitrogen contained in RA; foam degradation is therefore further promoted by the solubilisation of the gas phase into the liquid phase between the bubbles due to a high rate of interfacial mass transfer [38].

Furthermore, foam stability and bubble size distribution have a significant effect on foam rheological properties, and thus on its flow behaviour during injection [39].

In virtually every experiment performed, PEM produced either the longest DT or was similar to that of most stable PCFs generated using RA, indicating the best cohesive stability in the model and slower rate of degradation. The combination of the specific gas formulation and more importantly, the low mean bubble size (no bubbles greater than ~500 μm in diameter upon foam generation) and narrow bubble size distribution (see inset in Fig. 8a) [32] resulted in a lower rate of Ostwald ripening. In terms of clinical significance, the longer the foam DT in the vessel, the more contact time the polidocanol surfactant has with the endothelial lining of the vessel wall.

It has been demonstrated that the efficacy of the sclerosant is increased when the liquid sclerosant is formulated into a foam [40]. As a liquid solution introduced into the bloodstream, the sclerosant becomes diluted in the blood volume and contact with the vessel wall is limited. When in foam form, the sclerosant is present at the bubble interface at high surface area and the foam can fill the vessel lumen, displacing the blood volume and contacting the vessel lining more efficiently with lower concentrations of sclerosant required. A balance of stability is required however, in order that the foam does not break down before it can efficiently fill the vein but also that it will degrade soon after, and the component gases be absorbed into the bloodstream. This ensures bubbles do not persist and travel within the circulation where there is potential for them to become lodged to form gas emboli in small vessels in the brain. PEM offers an optimal balance of the cohesive stability characteristics of a nitrogen-containing foam providing for efficient vessel filling and piston effect to displace blood, coupled with bioabsorbable gas formulation in which the CO_2_ can dissolve in the blood and O_2_ sequestered by circulating haemoglobin.

Extrapolation of the results of our experiments to the clinical situation would suggest that if treating patients with larger varicosities, it may be of advantage to elevate the leg to a less extreme angle in order for the foam to more efficiently fill the vein and enable foam plug formation. Our model would also predict that the foam should be injected into the vein soon after it is formed, as all foams undergo some degree of degradation over time which will diminish their performance. The rate of injection is important, as we have seen that PCFs tend to require a faster rate of injection in order to ensure efficient foam plug formation in the vessel (0.45–0.55 mL foam/s or 9–11 s for a 5 mL foam injection). The administration of PEM is insensitive to this however, as foams with long DTs have been shown to be produced even when the rate of plug formation is low (0.26 mL foam/s or 19.2 s for a 5 mL foam injection). The use of PEM therefore has the advantage of consistency, reducing some of the inherent variability that arises from the use of PCF techniques.

## Conclusions

The biomimetic vein model has been shown to be useful for evaluating different foam formulations in terms of their cohesive stability and potential usefulness for varicose vein sclerotherapy. Stability of PCFs is affected by their method of manufacture, gas composition and liquid to gas ratio. CO_2_-containing foams have the advantage of gas solubility to aid in foam absorption in the bloodstream but are less stable and have shorter DTs in the vessel; a factor likely to affect the efficacy of the foam. The PEM device produces a foam that has the benefits of both a low-nitrogen gas formulation and enhanced foam stability for longer DT in the vein.

## Limitations and future work

There were some experimental limitations associated with this study. These are outlined below, and may represent the subject for future investigations:

- The stability of a foam plug in a vessel is likely to depend on the physical properties of the vessel, including wettability of its inner surface and surfactant adsorption to the surface. It likely that the PTFE vein model employed in the present study does not replicate those properties of veins. Future studies may be conducted on a second-generation vein model replicating more closely the physical and chemical properties of blood vessels.
- Experiments in this study have been conducted using a blood substitute (i.e., solution of glycerol in purified water). However, it has been shown that biological fluids (i.e., blood) have deactivation effects on sclerosants [41]. Therefore, further experiments should be performed to investigate the effect of the bio-physical properties of carrier fluids on the cohesiveness of sclerosing foams.
- Polidocanol was employed as a detergent sclerosant throughout these studies. However, a range of different sclerosing agents is available for application in foam sclerotherapy, and it is envisaged that different sclerosants (i.e., such as sodium tetradecyl sulphate or alcohol) may be investigated with our model in the future.

## Electronic supplementary material

Supplementary material 1 (DOCX 399 kb)

Supplementary material 2 (AVI 1231957 kb)

Supplementary material 3 (AVI 1216163 kb)

Supplementary material 4 (AVI 448560 kb)
