# Supplementary material for: The role of clinically-relevant parameters on the cohesiveness of sclerosing foams in a biomimetic vein model
Source: J Mater Sci Mater Med. 2015 Oct 8;26(11):258. doi: 10.1007/s10856-015-5587-z (PMC4598354; doi:10.1007/s10856-015-5587-z)
Supplement: Supplementary file 1 — Supplementary material 1 (DOCX 399 kb) [file 10856_2015_5587_MOESM1_ESM.docx]

**Supplementary Information for:**

**The role of clinically-relevant parameters on the cohesiveness of sclerosing foams in a biomimetic vein model**

*Dario Carugo^1,2*^, Dyan Ankrett^2^, Vincent O’Byrne^3^, David D I Wright^4^, Andrew L Lewis^3^, Martyn Hill^2,5^ and Xunli Zhang^1,5^*

*^1^* Bioengineering Science Research Group, Faculty of Engineering and the Environment, University of Southampton, Southampton SO17 1BJ, United Kingdom

*^2^* Electro-Mechanical Engineering Research Group, Faculty of Engineering and the Environment, University of Southampton, Southampton SO17 1BJ, United Kingdom

*^3^* Biocompatibles UK Ltd., Farnham Business Park, Weydon Lane, Farnham, Surrey GU9 8QL, United Kingdom

*^4^* BTG International Ltd., 5 Fleet Place, London EC4M 7RD, United Kingdom

*^5^* Institute for Life Sciences, University of Southampton, Southampton SO17 1BJ, United Kingdom

**Figure S1(a).** Varithena® (polidocanol injectable foam) 1% canister activation

Open the Bi-Canister pouch using a pair of scissors. Place canisters upright on a clean stable surface with the white oxygen canister on top (a).

Remove the safety clip by lifting one corner of the clip out. To begin the gas transfer, twist the canisters together clockwise (b) until they come to a stop and the small indicators/marks on the collars are aligned (c). You may hear a bubbling sound. While the canisters are activating, keep them upright on a clean flat surface for 1 minute. Twist the two canisters by turning them in the opposite direction (counterclockwise) as before (d) and pull straight up to separate the oxygen canister from the Varithena canister (e).

Remove the Varithena transfer unit from the blister pack and immediately place it on top of the blue Varithena canister (f). Gently rotate the Varithena transfer unit clockwise as indicated until it drops into the collar threads then twist the Varithena transfer unit (clockwise) until it reaches a stop (g). **The system is now activated and ready for use.**

**Figure S1(b):** Varithena® (polidocal injectable foam) 1% dispensing

Open a sterile 10 mL silicone-free syringe and connect it to the Varithena transfer unit as shown (a). Gently press down the Varithena transfer unit to begin producing foam (b). Using continuous pressure, allow the silicone-free syringe to fill between 3 mL and 5 mL. Release the pressure on the Varithena transfer unit and leave the syringe connected. Push the silicone-free syringe plunger in fully to discard its contents (c). Do not disconnect the syringe. Note: The foam will automatically be diverted into the waste chamber within the Varithena transfer unit. This process eliminates the small quantity of air in the syringe and Varithena transfer unit. While holding the silicone-free syringe plunger in place, gently press down on the Varithena transfer unit to begin the purge cycle (d).Visually inspect the ﬂowing foam inside the Varithena transfer unit to make sure the visible air bubbles have been expelled (less than 1 sec) before releasing the syringe plunger and allowing it to ﬁll to the desired volume (e). Draw up to 5 mL of foam into the syringe (f).

To replace the Varithena transfer unit, twist it counterclockwise then pull up to separate from the canister (g). Discard the old Varithena transfer unit and open a new Varithena transfer unit. Make sure not to touch the sterile underside of the Varithena transfer unit.

Important Note: Do not replace the Varithena transfer unit if the canister is to be stored for future use. The activated Varithena canister should always be stored with a Varithena transfer unit in place in the upright position at controlled room temperature. Replace the Varithena transfer unit just prior to its next use. Swab the uncovered shuttle with a fresh sterile alcohol wipe (g). Immediately place the new Varithena transfer unit on top of the Varithena canister (h). Gently rotate the Varithena transfer unit clockwise until it drops into the collar threads, then twist the Varithena transfer unit (clockwise) until it reaches a stop (h). **The Varithena canister is now ready for its next use.**

**Supplementary Video S1:**

This video shows the injection of 100% CO_2_ PCF (DSS method) in the biomimetic vein model with the corresponding real-time computational foam analysis dynamic plot.

**Supplementary Video S2:**

This video shows the injection of RA PCF (DSS method) in the biomimetic vein model with the corresponding real-time computational foam analysis dynamic plot.

**Supplementary Video S3:**

This video shows the injection of PEM in the biomimetic vein model with the corresponding real-time computational foam analysis dynamic plot.
